# Clinicians’, patients’ and carers’ perspectives on borderline personality disorder in Pakistan: A mixed methods study protocol

**DOI:** 10.1371/journal.pone.0286459

**Published:** 2023-06-02

**Authors:** Thea Lynne Hedemann, Muqaddas Asif, Huma Aslam, Aneela Maqsood, Ameer Bukhsh, Tayyeba Kiran, Umair Ahsan, Salman Shahzad, Juveria Zaheer, Steven Lane, Nasim Chaudhry, M. Ishrat Husain, M. Omair Husain

**Affiliations:** 1 Department of Psychiatry, University of Toronto, Toronto, ON, Canada; 2 Pakistan Institute of Living and Learning, Karachi, Pakistan; 3 Department of Psychiatry and Behavioral Sciences, Allama Iqbal Medical College and Jinnah Hospital, Lahore, Pakistan; 4 Fatima Jinnah Women University, Rawalpindi, Pakistan; 5 Institute of Clinical Psychology, University of Karachi, Karachi, Pakistan; 6 Centre for Addictions and Mental Health, Toronto, ON, Canada; 7 University of Liverpool, Liverpool, United Kingdom; Universidad Internacional de La Rioja, SPAIN

## Abstract

Borderline Personality Disorder (BPD) is a condition characterised by significant social and occupational impairment and high rates of suicide. In high income countries, mental health professionals carry negative attitudes towards patients with BPD, find it difficult to work with patients with BPD, and even avoid seeing these patients. Negative attitudes and stigma can cause patients to fear mistreatment by health care providers and create additional barriers to care. Patients’ self-stigma and illness understanding BPD also affects treatment engagement and outcomes; better knowledge about mental illness predicts intentions to seek care. The perspectives of mental health clinicians and patients on BPD have not been researched in the Pakistani setting and likely differ from other settings due to economic, cultural, and health care system differences. Our study aims to understand the attitudes of mental health clinicians towards patients with BPD in Pakistan using a self-report survey. We also aim to explore explanatory models of illness in individuals with BPD and their family members/carers using a Short Explanatory Model Interview (SEMI). The results of this study are important as we know attitudes and illness understanding greatly impact care. Results of this study will help guide BPD-specific training for mental health clinicians who care for patients with BPD and help inform approaches to interventions for patients with BPD in Pakistan.

## Introduction

Borderline Personality Disorder (BPD) is a condition characterised by affect instability, impulsivity, low distress tolerance, and self-harming behavior [1]. Not only is BPD associated with significant social and occupational impairment, affected individuals also have reduced life expectancy and up to 10% of individuals with BPD die by suicide [1]. Data from the United States demonstrates that BPD affects 1–2% of the general population, while accounting for 10% of psychiatric outpatients and 20% of psychiatric inpatients [1]. In Pakistan, the exact prevalence of BPD remains unknown; however, survey data indicates that up to 18% of the Pakistani population may exhibit BPD traits [2]. Effective treatment often involves targeted psychotherapy, such as dialectical behavior therapy (DBT), which typically involves at least weekly contact with clinicians for up to a year [3]. Accessing treatment can reduce suicidal behavior, self-injury, improve interpersonal functioning, and reduce core BPD symptoms [4].

The majority of BPD research has been conducted in high-income, Western countries like the United States, the United Kingdom, and Canada despite BPD being prevalent globally [4,5]. Research from these settings is not necessarily translatable to other contexts with significant cultural, economic and health system differences. Pakistan presents a unique context for conducting this research on BPD. It is a lower-middle-income country that features diverse ethnic groups, religious communities, and subcultures, with individuals holding unique illness beliefs and values [6]. For instance, beliefs attributing mental illnesses to supernatural causes are common [7]. Factors such as education level, socioeconomic status, and exposure to mental health information may influence these explanatory models.

Despite BPD being highly treatable, accessing treatment is often difficult. Poor illness understanding and stigma serve as major barriers to care and recovery [8–10]. Negative illness attitudes are associated with reduced self-efficacy, increased hopelessness, low self-esteem, and poorer quality of life [7]. In Pakistan, mental health is more stigmatized than physical health conditions, and explanatory models of mental illness rooted in supernatural causes contribute to stigma [7]. Enhanced mental health literacy in patients and caregivers can improve intentions to seek care, ultimately contributing to improved treatment outcomes and recovery [1,11].

Clinicians are not exempt from stigma [7]. Studies in high-income settings have revealed that clinicians harbor negative attitudes towards BPD patients, often finding it difficult to work with them or even avoiding them altogether [12–14]. Such attitudes can exacerbate patients’ fear of mistreatment by healthcare providers and further hinder access to care [15]. A study conducted in Italy demonstrated that increased exposure to patients with BPD was positively associated with caring attitudes, suggesting that more frequent contact may help to challenge and overcome preconceived beliefs about these patients [13]. They also found that newer clinicians had more caring attitudes towards BPD patients, which may be related to the recency of their education. Clinicians with more years of experience may have attitudes reflective of an earlier era, where outcomes for BPD patients were less hopeful. Notably, variations in clinician attitudes can result from differences in professional backgrounds and may be linked to gaps in mental health education [13]. For example, the same study showed that nurses and social workers held more stigmatizing attitudes towards patients with BPD than psychologists and psychiatrists, who often receive more BPD specific training. While Pakistani clinicians are suspected to hold attitudes towards BPD patients similar to those in high-income settings like Italy, these findings have yet to be confirmed in Pakistan.

The present study aims to investigate the attitudes of mental health clinicians in Pakistan towards patients with BPD. We also aim to examine explanatory models of illness in individuals with BPD and their family members/carers through semi-structured interviews. We hypothesize negative attitudes towards patients with BPD among clinicians are prevalent, with intergroup differences based on occupational backgrounds and clinical experience. In the patient and family members/carers component of our study, we hypothesize that the explanatory models of BPD among patients and their family members/carers will vary across distinct subcultures in Pakistan, with a limited overall understanding of the disorder’s etiology, prognosis, and treatment.

The findings of this study hold significant implications, as both clinician attitudes and patients’ and families’ illness understanding can substantially influence care [15]. By identifying disparities among clinicians, tailored interventions and educational programs can be developed to target specific groups, such as new graduates or occupational groups. Gaining deeper insights into patients’ illness understanding can inform efforts to reduce stigma and shape future interventions for individuals with BPD in Pakistan. Moreover, by detecting subcultural variations, tailored interventions and educational programs can be developed that respect the unique cultural context of each subpopulation and enhance recovery outcomes.

To our knowledge, no prior studies have examined explanatory models of BPD in patients and their family members/carers, or clinicians’ attitudes toward BPD in Pakistan. This research, therefore, seeks to fill these knowledge gaps and contribute to the development of culturally sensitive and effective mental health care strategies, ultimately improving outcomes for individuals affected by BPD in the region.

## Materials and methods

### Aims

The goal of this study is to explore the attitudes of mental health clinicians and explanatory models of patients and family members/carers on BPD in Pakistan. The attitudes of mental health clinicians will be explored through a self-report survey while patient and family members/carers’ perspectives will be explored using the Short Explanatory Model Interview (SEMI) [16].

Aim 1: To examine attitudes of mental health clinicians towards patients with BPD in Pakistan.

We hypothesize that nearly half of clinicians carry negative attitudes towards patients with BPD. Our prevalence estimate has been informed by previously published literature albeit from high income countries [12].

Aim 2: To evaluate the differences in "Overall Caring Attitudes" scores among various occupational subgroups (i.e., physicians, resident physicians, nurses, psychologists, social workers), as well as the influence of overall years of clinical experience, and clinical experience with patients with BPD. We hypothesize that greater clinical exposure to patients with BPD will be positively associated with higher "Overall Caring Attitudes" scores. Conversely, higher number of years in practice will be negatively associated with "Overall Caring Attitudes" scores. Additionally, we anticipate significant score variations between the occupational subgroups, with physicians and psychologists having higher “Overall Caring Attitudes” than social workers and nurses. These hypotheses are informed by previous literature [13].

Aim 3: To explore and describe the explanatory models of BPD held by patients and their family members/carers in the Pakistani context. We hypothesize that most participants will utilize multiple explanatory models to understand their or their loved one’s illness, with supernatural beliefs being the most common. We expect that biological, psychological (e.g., stress), social (e.g., interpersonal), and environmental models will be less prevalent and that awareness of effective psychological treatments for BPD will be low (<50% of participants). Our hypotheses draw on previous research on severe mental disorders in South Asian populations, which favored spiritual explanations over biological, psychological, or social models [17]. Additionally, we anticipate differences in explanatory models among subcultures (our eight participating sites) within Pakistan, with subcultures exhibiting lower education levels (based on local literacy rates) demonstrating higher prevalence of spiritual explanatory models.

### Design

This is a cross-sectional study that will employ a mixed methods approach to explore the attitudes of mental health clinicians and the illness understanding of patients with BPD and their family members/carers on BPD in Pakistan. Quantitative data related to the attitudes of mental health clinicians will be explored through a self-report survey while qualitative data related to patient and family members’/carers will be explored using a SEMI, which includes a case vignette.

Clinician attitudes will be explored through an electronic survey or paper-based survey to a purposive sample of clinicians (n = 500; psychiatrists, psychiatry residents, social workers, nursing staff, occupational therapists). An introduction to the project and consent will be obtained prior to agreeing to participate and completing the survey.

Through purposive sampling we will recruit at total of 50 patients and 50 family members/carers to share perspectives of illness using the SEMI. Participants will be recruited from both inpatient and outpatient settings. Those patients and family members/carers that are potentially eligible and provide verbal permission to their clinical teams will then be approached by our RAs directly. RAs will then explain the study and go through the patient information leaflet. The RAs will obtain informed consent from patients and carers prior to participants being enrolled in the study.

We will also advertise with posters in these clinics so patients and carers can reach out directly if they are interested in participating. We will also advertise on the Pakistan Institute for Living and Learning social media platforms and website.

### Study setting

There are eight participating sites across Pakistan: Rawalpindi, Lahore, Karachi, Quetta, Hyderabad, Peshawar, and Nawabshah. We have research assistants (RAs) based at all these sites. Local clinical teams will be approached by our RAs to screen potential participants for eligibility. We will be able to connect with participants through these sites’ inpatient and outpatient psychiatry departments. Clinicians from these sites will be recruited for participation in the survey component.

### Participants

#### Clinician participant inclusion criteria

- Provide informed consent
- Greater than one year of professional experience in mental health
- Psychiatrist, psychiatry resident, psychologist, nurse, social worker
- Currently working in mental health settings (outpatients or inpatients, including community mental health centers, or day programming)
- Participants must be able to read and respond to the survey in either English or Urdu.

#### Clinician participant exclusion criteria

- There are no specific exclusion criteria for clinician participants

#### Patient participant inclusion criteria

- DSM-V criteria for BPD confirmed by SCID interview
- Participants will be 18 years or older.
- Provide informed consent to participate in the study

#### Patient participant exclusion criteria

- Participants with co-morbid bipolar affective disorder, type I
- Participants with a co-morbid primary psychotic disorder

#### Family member/carer participant inclusion criteria

- Family member/carer of an individual with BPD who is aged 18 years or older.
- Willing to provide informed consent to participate in the study

#### Patient participant exclusion criteria

- Family member/carer of an individual with BPD and co-morbid bipolar affective disorder, type I or primary psychotic disorder

We excluded providers with less than 1 year experience as based on existing literature exploring clinician attitudes [12,13]. We wanted our results to reflect clinicians with at least some clinical exposure which could help inform their attitudes. Anxiety and depression are highly prevalent within Pakistanis and are common co-morbidities with borderline personality disorder [2]. For these reasons, we did not exclude patients with these comorbidities. Pragmatically, excluding individuals with these comorbidities would likely impact the feasibility of recruitment. We did not want participants’ explanatory models to be shaped by other severe mental illnesses like psychotic disorders and/or bipolar disorder. The SCID-5-PD interview tool is most appropriate for use in participants 18 and older [18]. This shaped our rationale for only including patients 18 years or older. With this minimum age for patients, we only included family members/carers of adult patients to maintain consistency.

### Assessments and instruments

#### Clinician survey on clinician attitudes towards BPD

A self-report questionnaire (available in English and Urdu) will be distributed to mental health clinicians at eight participating centres in Pakistan. Surveys will be distributed electronically and/or in person. Clinicians will be asked to assess their attitudes by rating their agreement with the items using a Likert scale (1 = strongly agree, 7 = strongly disagree). Demographic data, such as gender and occupation, will also be collected. Additionally, we will inquire about the number of BPD patients cared for in the past year and the clinician’s years of experience working with psychiatric patients.. This questionnaire has been adapted from the study conducted by Black et al. (2011) in the United States, and later replicated in Italy by Lanfredi et al. (2021) [12,13]. This survey was chosen through expert consensus, due to its relevance and applicability to the research objectives. It is important to note that there is limited validity data or gold standard measures for assessing BPD attitudes, particularly in the Pakistani context. In prior research, internal consistency was found to have a Cronbach’s alpha of 0.68, suggesting moderate consistency in measuring the underlying construct of caring attitudes towards BPD patients [13].

#### The Structured Clinical Interview for DSM-5 Personality Disorders (SCID-5-PD)

The SCID-5 is a semi-structured interview used to accurately diagnose DSM-V conditions for research and clinical purposes [18], The SCID-5-PD is a specific version used to evaluate personality disorders [18]. SCID interviews of patients will be conducted by psychologists who have received training in SCID-5-PD. There will be ongoing concordance rating sessions under the supervision of senior clinical psychologists and psychiatrists to ensure the accuracy of codes.

#### Short Explanatory Model Interview (SEMI)

The Semi-Structured Explanatory Model Interview (SEMI) is an interview methodology that incorporates a blend of open-ended and closed-ended questions, organized into five sections: personal background and demographics, the nature of the presenting issue, help-seeking behavior, interactions with healthcare providers or healers, and beliefs regarding mental illness [16]. The SEMI aims to elucidate patients’ perspectives on their conditions through open-ended inquiries. This comprehensive tool is designed for application in clinical environments and research and has been extensively implemented in India and Pakistan for a range of mental health disorders [19–22]. Conducted in Urdu, the SEMI will be administered and coded by research assistants who have undergone specialized training, with a senior clinical psychologist available to address any ambiguous responses.

The SEMI is employed with two critical components: evaluating participants’ comprehension of their own illness or their family members’ illness (non-clinical vignette) and presenting a clinical vignette depicting a patient with BPD. The BPD vignette, developed by the study’s authors, delineates the core symptoms of BPD in accordance with the DSM-5 criteria. To ensure validity, the vignette has been reviewed and refined by other psychiatrists, avoiding technical language, and omitting explicit reference to BPD. Both the non-clinical and clinical vignettes play a role in exploring participants’ beliefs and understanding of BPD. While the non-clinical vignette captures participants’ personal experiences with BPD, the clinical vignette provides an opportunity for them to express their broader understanding of the disorder. With the participants’ consent, trained research assistants will record and transcribe the SEMI interviews verbatim. The resulting data will be subjected to both qualitative and quantitative analysis.

### Sample size and data analysis

500 clinicians will be included in the survey using purposive sampling. Previous data shows that 47% of clinicians would rather “avoid caring for a BPD patient” [12]. Using this as our estimated prevalence of negative attitudes towards BPD, our minimum calculated sample size is 383 with a 95% confidence level and margin of error of 5%. We have increased our sample size to 500 clinicians to allow for drop out or non-participation [12,13].

To assess whether clinicians harbor negative attitudes towards patients with BPD, we will calculate the percentage of participants who endorse item 1, "If I had a choice, I would prefer to avoid caring for a BPD patient," on the survey. This approach will enable us to quantify the prevalence of negative attitudes among the participating clinicians, as employed in previous studies [12].

To calculate an “Overall Caring Attitudes” score, each Likert response will be provided a value between 1–7, where “strongly agree” would equal 1, and “strongly disagree” would equal 7. The mean Likert scale scores as a sum of all the values for each survey question with standard errors will be calculated. Scores for questions 2, 4, 7, 9, 10, 11, and 13 on the survey will be reversed prior to determining their means such that higher mean score represents more positive attitudes. The “Overall Caring Attitudes” score will be calculated by deriving the overall mean value from the Likert Questions. Any questionnaires with a small item correlation, will be excluded from the overall mean value calculation.

All quantitative results will be analysed using SPSS (version 17 for Windows) [23]. We will use means and standard deviations with corresponding confidence intervals. One-way ANOVA will be used to investigate differences in “Overall Caring Attitudes” between clinician groups (i.e. physicians, resident physicians, nurses, psychologists, social workers). An ordinal logistic regression model will be used to assess for the effect of predictors including occupation, number of patients with BPD cared for in the past year (0–2, 3–9, 10–24, or >25), and years of clinical experience (0–4, 5–10, 11–19, or >20) on “Overall Caring Attitudes”. Covariates will include gender and practice location (i.e. inpatient, outpatient, site).

A sample size of 50 patients and 50 family members/carers for the SEMIs was determined based on previous literature. Prior qualitative clinical research using semi-structured interviews has demonstrated that six to seven semi-structured interviews are needed to achieve thematic saturation [24]. With eight participating sites, our sample size ranging from 48 to 56 would achieve data saturation at each site. A large overall and diverse sample size will enhance the generalizability of the results [25].

We will employ directed content analysis to analyze the open-ended responses from the SEMI [26]. This approach uses the SEMI’s established framework of categories and codes related to its open-ended questions for data analysis. First, researchers will read the textual data provided by participants for each category, identifying the text that corresponds to the predetermined codes. Then, experienced clinical psychologists, trained in using SEMI, will review the identified texts to ensure they properly align with the respective predetermined categories and codes. Any data that cannot be coded with the initial coding scheme will be further analyzed to determine if they represent a new category or a subcategory of an existing code.

Our third aim of the study will be achieved by analyzing the frequency of these categorical responses. Results from all sites will be summarized to provide generalizable results about the explanatory models of BPD in Pakistan. Thematic differences between sites will be described as subcultural differences.

### Data management

For data security, consent forms and paper copies of assessment tools having any identifying information such as name, address or phone number of participants will all be stored in locked filing cabinets in a secure office. All computerised data will be encrypted and password protected.

### Safety considerations

This observational study poses minimal risk to participants. Patient participants may feel uncomfortable answering some questions during the interview process, particularly ones about their own mental health; to address this, the consent process will inform patients about sensitive topics and their right to stop participating at any time. Discussing distressing topics can result in negative moods and stress but remains at low risk to cause harm. To mitigate any risk, we will forewarn patients about the topics of self-harm, trauma, and suicide in the consent form and emphasis they can stop participating at any time. Project manager contact information and a 24/7 helpline will be provided during the consent process. Information on local mental health support and emergency medical services will also be available.

We also recognize that patients with BPD may experience suicidal ideation or thoughts of self-harm. The Pakistan Institute of Living and Learning has established risk protocols in place for acute safety concerns. Trained researchers experienced in mental health will conduct the study, ensuring sensitive questioning and appropriate support if distress arises. If a participant becomes distressed, researchers will follow up with a risk assessment and provide a crisis plan. Participants will be advised to contact the principal investigator, a helpline, or their local mental health centre. Free psychological services will be offered by trained professionals if needed.

### Ethical considerations

This project has received ethics permission from the National Bioethics Committee of Pakistan (Ref: No.4-87/NBC-758/22/1816). All members of the research team will comply with the International Conference on Harmonization Good Clinical Practice (ICH-GCP) guidelines [27]. All research staff are trained on Good Clinical Practices (GCP) [27]. The information provided by the participants will be kept confidential and authorization will be required prior to access. Participants identifying information will not be published. Informed consent forms will be in the local language. Those, who are not able to read and write, will be provided with verbal information (in the local language) that will encompass all aspects of the participant written informed consent form. The consent form will then be signed by the participants and a caregiver will co-sign alongside the participant’s signature. However, if the participant cannot write, the participant’s caregiver will sign alongside the participant’s thumb print. The participant informed consent form will include details of the purpose of the study; the opportunity to ask questions; voluntary participation in the study and the right to withdraw from the study at any time, for any reason without impact on the care they receive; as well as privacy and confidentiality. All participants can decide to participate right away if they want to, or they will be given at least 48 hours to read and discuss queries with family or the research team before a decision for participation is made.

### Duration of the project

Timeline

- Project was approved by National Bioethics Committee of Pakistan April 7 2022
- Training of research assistants for conducting SEMI interviews completed December 2022 (at all 8 participating sites)
- Training of research assistants for SCID interviews by March 2023
- Data collection for survey and SEMI April—May 2023.
  ○ Aiming for 2 completed SEMI interviews per week at each site
  ○ Aiming for 10 completed surveys per week at each site
- Complete data analysis and develop manuscript by June 2023
- Review by co-authors submit for publication by July 2023.

We have site leads assigned for all 8 participating sites that will help with recruitment and management of the research assistants at each site. Data collection has not started.

## Discussion

### Expected outcomes of the study

This is the first study exploring clinician attitudes and patients and family members/carers’ illness understanding of BPD in Pakistan. We anticipate that most clinicians carry negative attitudes towards patients with BPD and that there will be differences based on occupational backgrounds and experience levels. We also anticipate that that patient and family members/carers have varied explanatory models of BPD, and their general understanding of BPD and treatment is limited. The results of this study are important as we know clinician attitudes and illness understanding can greatly impact care [15]. Results of this study can help identify current barriers, like sigma, that may be impeding care for patients with BPD in Pakistan. We hope our results will inform BPD-specific training for mental health clinicians, guide patient education strategies, and influence interventions for patients with BPD in Pakistan.

### Dissemination of results and publication policy

Study outcomes will be published as widely as possible in peer reviewed and non-peer reviewed journals and will be presented at local, national, and international conferences. Thea L Hedemann plans to lead the dissemination of results with the PILL team.

Our results will also be shared publicly through avenues like the PILL newsletter, blogposts, website, and social media pages to reach both the Pakistani community and the global mental health research community. Our results may also be shared at one of the several annual events organized by PILL to raise awareness about the importance of mental health across Pakistan. Our results may also be added to awareness seminars in educational institutions, organizations and in the wider community that are facilitated in collaboration with PILL.

### Problems anticipated

We are recruiting from eight different sites across Pakistan, all of which have their own subcultures, education level, and healthcare system differences. The diversity in participants means that our results may be heterogenous and common themes in our qualitative data may be more difficult to identify. Nonetheless, it is important to recruit participants from across Pakistan to enhance the ecological validity of our study.

Language could be a potential barrier for participants completing the survey. For this reason, we plan to distribute the survey both in English and Urdu to increase the response rate. We have chosen to conduct the SEMI in the national language, Urdu, despite the many languages spoken in Pakistan; this is a limitation of our translational expertise.

## Supporting information

S1 File SEMI for patients. (DOCX)

S2 File SEMI for family members/carers. (DOCX)

S3 File Survey. (PDF)
