# Supplementary material for: Clinicians’, patients’ and carers’ perspectives on borderline personality disorder in Pakistan: A mixed methods study protocol
Source: PLoS One. 2023 Jun 2;18(6):e0286459. doi: 10.1371/journal.pone.0286459 (PMC10237402; doi:10.1371/journal.pone.0286459)
Supplement: S2 File — (DOCX) [file pone.0286459.s002.docx]

***The Short Explanatory Model Interview (SEMI)***

1. INTRODUCTION:

“Thank you for agreeing to talk about your health. I would like to ask you some questions about your health and how it affects you. The questions have already been written out so it will not sound like a normal interview and some things may not have much to do with your situation. I would like to stress that all your answers will be strictly confidential.”

| 1.1-3 | Record number |  | rec |
| --- | --- | --- | --- |
| 1.4-9 | Date of interview |  | seen |
| 1.10 | Gender |  | sex |
| 1.11-12 | Age <How old were you on your last birthday?> |  |  |

1. HEALTH & ILLNESS: CURRENT HEALTH STEM:
   1. I would like to ask you about your <last> visit to the doctor

What < have you come / did you go about >? (list up to 3 reasons).

| 2.1-2.2 |  | problem1 |
| --- | --- | --- |
| 2.3-2.4 |  | problem2 |
| 2.5-2.6 |  | problem3 |

HEALTH OVER LAST YEAR STEM.

b. Over the past year have you had any illness or health problems? (list up to three).

| 2.7-2.8 |  | Year1 |
| --- | --- | --- |
| 2.9 -2.10 |  | Year2 |

a. What do you call these problems? Probe: If you had to give them names, what would they be?

| 2.13-2.14 |  | Name1 |
| --- | --- | --- |
| 2.15-2.16 |  | Name2 |
| 2.17.18 |  | Name3 |

d. When did you first notice <specify identified problem>? Probe: how long ago was it, when did it start?

| 2.19-2.20 |  | Onset1 |
| --- | --- | --- |
| 2.21-2.22 |  | Onset2 |
| 2.23-2.24 |  | Onset3 |

e. Why do you think these problems started when they did?

| 2.25-2.26 |  | Why1 |
| --- | --- | --- |
| 2.27-2.28 |  | Why2 |
| 2.29-2.30 |  | Why3 |

1. Is there anything you have or haven’t done that has caused this? Probe for example.

| 2.31 |  | internal |
| --- | --- | --- |

1. Is there anything anyone else has done or not done that has caused this? Probe e.

| 2.32 |  | external |
| --- | --- | --- |

1. So who or what is the cause of you getting this?

| 2.33 |  | Intext |
| --- | --- | --- |

1. Do you believe that someone or something can <perform magic / put a spell on other people (seek examples)?

| 2.34 |  | spells |
| --- | --- | --- |

1. PERCEIVED SEVERITY
   1. How serious are your problems?

| 3.1 |  | Serious1 |
| --- | --- | --- |
| 3.2 |  | Serious2 |
| 3.3 |  | Serious3 |

- 1. What do you most fear about these problems?

| 3.4 |  | Fear1 |
| --- | --- | --- |
| 3.5 |  | Fear2 |
| 3.6 |  | Fear3 |

- 1. Why did you go to the doctor? Probe: Had it got worse? In what way? Were you afraid what it might be, did other people advise you to go?

| 3.7 |  | Why come |
| --- | --- | --- |

1. EXPECTATIONS OF / SATISFACTION WITH MEDICAL CARE
   1. What do/did you hope to gain from seeing your doctor? What do/did you want the doctor to do?

| 4.1 |  | Expect1 |
| --- | --- | --- |
| 4.2 |  | Expect2 |
| 4.3 |  | Expect3 |

- 1. Have you asked the doctor about these problems?

| 4.4 |  | askgp |
| --- | --- | --- |

- 1. What did the doctor do about these problems?

| 4.5 |  | Gpact1 |
| --- | --- | --- |
| 4.6 |  | Gpact2 |

| 4.7 |  | Gpact3 |
| --- | --- | --- |

- 1. Was it useful talking to the doctor about your problems? Can you say why?

| 4.8 |  | satisfy |
| --- | --- | --- |

- 1. Was there anything about your treatment you are unhappy about?

| 4.9 |  | unhappy |
| --- | --- | --- |

1. ACTIVITIES AND FUNCTIONING
   1. What are the main difficulties your problems have caused you (list up to 3)?

| 5.1-5.2 |  | Difs1 |
| --- | --- | --- |
| 5.3-5.4 |  | Difs2 |
| 5.5-5.6 |  | Difs3 |

- 1. Which parts of your body are most affected by your problems (list up to 3)?

| 5.7 |  | Body1 |
| --- | --- | --- |
| 5.8 |  | Body2 |
| 5.9 |  | Body3 |

c. How have you been affected emotionally by what you’ve described? (give e.g.)

| 5.10 |  | emotion |
| --- | --- | --- |

d. Have these problems stopped you getting about as well as you used to? (e.g.)

| 5.11 |  | mobile |
| --- | --- | --- |

e. Have these problems affected your social life? (give examples)?

| 5.12 |  | Social |
| --- | --- | --- |

1. Have these problems affected your home life? (give examples)?

| 5.13 |  | Family |
| --- | --- | --- |

1. Have these problems affected how you get on with people in general? (give e.g.)

| 5.14 |  | Relate |
| --- | --- | --- |

1. Has your work been affected (how?)

| 5.15 |  | Work |
| --- | --- | --- |

1. OTHER HEALTH BEHAVIOUR
   1. Have you asked for advice from anyone else about these problems? Probe: hospital, pharmacist, friends, family, church, healers, osteopaths etc.

| 6.1 |  | advice |
| --- | --- | --- |

- 1. Has anyone else apart from your doctor given you any Rx or advice about this?

| 6.2 |  | Nongp |
| --- | --- | --- |

- 1. Are you treating yourself for the problem?

| 6.3 |  | Self |
| --- | --- | --- |

- 1. If so how?

| 6.4 |  | How |
| --- | --- | --- |

- 1. Are you taking any medication? (what is it)

| 6.5-6.6 |  | Meds1 |
| --- | --- | --- |
| 6.7-6.8 |  | Meds2 |
| 6.9-6.10 |  | Meds3 |

- 1. Are you taking any other cures or remedies?

| 6.12 |  | cures |
| --- | --- | --- |

- 1. Do you smoke? (how much)

| 6.13 |  | cigs |
| --- | --- | --- |

- 1. Do you drink alcohol (how much)?

| 6.14 |  | alcohol |
| --- | --- | --- |

- - 1. What about any <street/recreational> drugs (what? give examples)

| 6.15 |  | drugs |
| --- | --- | --- |

1. **VIGNETTE:**

Read out “You’ve been kind enough to tell me about yourself and your visit to the doctor. Finally, I’d like to ask your opinion about some other peoples’ visit to the doctor. I’d like to read a short account of their problems and then ask you a few questions about them.”

Ms. K was taken away from her family at the age of 10 because her parents were unable to care for her. While growing up with distant family members, she was constantly yelled at and physically abused by them. She got into trouble for her anger outbursts with other children in school and in the neighbourhood. Ms. K often felt alone, and she began to cope by cutting her thighs with a razor and impulsively binge-eating. She felt empty most of the time and often thought about overdosing on pills at home to end her life.

Ms. K went away for postsecondary school, where she started to make friends and felt she was making a fresh start. She often impulsively spent money on presents for her friends she couldn’t afford, hoping to keep her friends close. After finding out that her friends did not include her in a birthday celebration, Ms. K sent her friends angry text messages that she later regretted. When they ignored her repeated phone calls, she felt completely abandoned and thought life was not worth living. Ms. K then attempted to end her life by overdosing on paracetamol.

After graduating from school, she continued to have difficulties with sadness, emptiness, and anger. Her emotions would fluctuate throughout the course of a day. Despite her efforts to make new friendships, others found her to be intense and this would lead to relationships breaking down. Her identity and self-image continued to be unstable throughout her life.

While working at her retail job, she would get easily overwhelmed when her boss criticized her. She continued to turn to cutting herself and binge-eating to cope with her strong emotions. At 23, she attempted suicide again after being fired for getting mad at her boss in front of customers.

- 1. What if anything is her problem?

7.1-7.2

what1

b. Does she have an illness. If yes, what is it?

7.3-7.4

illness1

1. What are the causes of her problems?

7.5-7.6

cause1

1. What should she do about it.?

7.7-7.8

action1

1. What should the doctor do about it?

7.9-7.10

gpdo1

1. Finally, is there anything else about your recent trip to the doctor or health we haven’t talked about you would like to say? (continue on extra sheets if needed)

8.1

note

Thank you
