# Supplementary material for: Clinicians’, patients’ and carers’ perspectives on borderline personality disorder in Pakistan: A mixed methods study protocol
Source: PLoS One. 2023 Jun 2;18(6):e0286459. doi: 10.1371/journal.pone.0286459 (PMC10237402; doi:10.1371/journal.pone.0286459)
Supplement: S3 File — (PDF) [file pone.0286459.s003.pdf]

Survey for "*Perspectives on Borderline Personality Disorder in Pakistan: A Clinician Survey and a Short Explanatory Model Interview (SEMI) of Patients*"

This survey will also be available in Urdu for participants.

Age: __
Gender
- o Male
- o Female

Estimated number of BPD patients cared for in the past year
- o 0-2
- o 3-9
- o 10-24
- o >25

Years in mental health
- o 0-4 years
- o 5-10 years
- o 11-19 years
- o >20 years

Primary Work setting
- o Academic Hospital Inpatient Setting
- o Academic Hospital Outpatient Setting
- o Community Inpatient Setting
- o Community Outpatient Setting
- o Other: _____________

Work Location
- o Karachi
- o Lahore
- o Peshawar
- o Rawalpindi
- o Multan
- o Quetta
- o Hyderabad
- o Other: ___________

Occupation
- o Psychiatry resident
- o Psychiatrist
- o Social work
- o Staff nurse
- o Psychologist
- o Other: __________

Please rate how much you agree or disagree with the following statements.

1. If I had a choice, I would prefer to avoid caring for a BPD patient.

[ ] Strongly Disagree [ ] Disagree [ ] Somewhat Disagree [ ] Neutral [ ] Somewhat Agree [ ] Agree [ ] Strongly Agree

2. I feel professionally competent to care for BPD patients.
   [ ] Strongly Disagree [ ] Disagree [ ] Somewhat Disagree [ ] Neutral[ ] Somewhat Agree [ ] Agree [ ] Strongly Agree

3. BPD patients intentionally manipulate others.
   [ ] Strongly Disagree [ ] Disagree [ ] Somewhat Disagree [ ] Neutral [ ] Somewhat Agree [ ] Agree [ ] Strongly Agree

4. Providing care to a patient with BPD is highly satisfying.
   [ ] Strongly Disagree [ ] Disagree [ ] Somewhat Disagree [ ] Neutral [ ] Somewhat Agree [ ] Agree [ ] Strongly Agree

5. It is easy for me to stereotype patients with BPD.
   [ ] Strongly Disagree [ ] Disagree [ ] Somewhat Disagree [ ] Neutral [ ] Somewhat Agree [ ] Agree [ ] Strongly Agree

6. I dislike BPD patients.
   [ ] Strongly Disagree [ ] Disagree [ ] Somewhat Disagree [ ] Neutral [ ] Somewhat Agree [ ] Agree [ ] Strongly Agree

7. BPD is an illness that causes symptoms that are distressing to the BPD individual.
   [ ] Strongly Disagree [ ] Disagree [ ] Somewhat Disagree [ ] Neutral [ ] Somewhat Agree [ ] Agree [ ] Strongly Agree

8. Most staff currently lack the expertise to adequately care for BPD patients.
   [ ] Strongly Disagree [ ] Disagree [ ] Somewhat Disagree [ ] Neutral [ ] Somewhat Agree [ ] Agree [ ] Strongly Agree

9. I believe the BPD patient has low self-esteem.
   [ ] Strongly Disagree [ ] Disagree [ ] Somewhat Disagree [ ] Neutral [ ] Somewhat Agree [ ] Agree [ ] Strongly Agree

10. Some medications are very effective in helping patients with BPD.
    [ ] Strongly Disagree [ ] Disagree [ ] Somewhat Disagree [ ] Neutral [ ] Somewhat Agree [ ] Agree [ ] Strongly Agree

11. I feel I can make a positive difference in the lives of BPD patients.
    [ ] Strongly Disagree [ ] Disagree [ ] Somewhat Disagree [ ] Neutral [ ] Somewhat Agree [ ] Agree [ ] Strongly Agree

12. The prognosis for BPD treatment is hopeless.
    [ ] Strongly Disagree [ ] Disagree [ ] Somewhat Disagree [ ] Neutral [ ] Somewhat Agree [ ] Agree [ ] Strongly Agree

13. Some psychotherapies are very effective in helping patients with BPD.

[ ] Strongly Disagree [ ] Disagree [ ] Somewhat Disagree [ ] Neutral [ ] Somewhat Agree [ ] Agree [ ] Strongly Agree

14. BPD is not a valid diagnosis – BPD symptoms are really due to other disorders.
[ ] Strongly Disagree [ ] Disagree [ ] Somewhat Disagree [ ] Neutral [ ] Somewhat Agree [ ] Agree [ ] Strongly Agree

15. I would like more training in the management of BPD patients.
[ ] Strongly Disagree [ ] Disagree [ ] Somewhat Disagree [ ] Neutral [ ] Somewhat Agree [ ] Agree [ ] Strongly Agree

This survey has been adapted from previous studies.[1,2]

References:

1. Black DW, Pfohl B, Blum N, McCormick B, Allen J, North CS, et al. Attitudes Toward Borderline Personality Disorder: *A Survey of 706 Mental Health Clinicians*. CNS Spectr. 2011 Mar;16(3):67–74.

2. Lanfredi M, Ridolfi ME, Occhialini G, Pedrini L, Ferrari C, Lasalvia A, et al. Attitudes of Mental Health Staff Toward Patients With Borderline Personality Disorder: An Italian Cross-Sectional Multisite Study. Journal of Personality Disorders. 2019 Feb 20;1–16.
